# Assessment of Temperature Rise and Time of Alveolar Ridge Splitting by Means of Er:YAG Laser, Piezosurgery, and Surgical Saw: An Ex Vivo Study

**DOI:** 10.1155/2016/9654975

**Published:** 2016-11-10

**Authors:** Jacek Matys, Rafał Flieger, Marzena Dominiak

**Affiliations:** ^1^Private Dental Healthcare, Ul. Lipowa 18, 67-400 Wschowa, Poland; ^2^Private Dental Healthcare, Ul. Nacławska 11, 64-000 Kościan, Poland; ^3^Department of Dental Surgery, Medical University of Wroclaw, Ul. Krakowska 26, 50-425 Wroclaw, Poland

## Abstract

The most common adverse effect after bone cutting is a thermal damage. The aim of our study was to evaluate the bone temperature rise during an alveolar ridge splitting, rating the time needed to perform this procedure and the time to raise the temperature of a bone by 10°C, as well as to evaluate the bone carbonization occurrence. The research included 60 mandibles (*n* = 60) of adult pigs, divided into 4 groups (*n* = 15). Two vertical and one horizontal cut have been done in an alveolar ridge using Er:YAG laser with set power of 200 mJ (G1), 400 mJ (G2), piezosurgery unit (G3), and a saw (G4). The temperature was measured by K-type thermocouple. The highest temperature gradient was noted for piezosurgery on the buccal and lingual side of mandible. The temperature rises on the bone surface along with the increase of laser power. The lower time needed to perform ridge splitting was measured for a saw, piezosurgery, and Er:YAG laser with power of 400 mJ and 200 mJ, respectively. The temperature rise measured on the bone over 10°C and bone carbonization occurrence was not reported in all study groups. Piezosurgery, Er:YAG laser (200 mJ and 400 mJ), and surgical saw are useful and safe tools in ridge splitting surgery.

## 1. Introduction

The important condition for predictable and aesthetic implantation is the availability of sufficient surrounding and supporting hard and soft tissues [1]. Due to the significant loss of alveolar bone additionally surgical procedures are necessary [2]. Augmentation of the resorbed alveolar crest can be achieved, for example, with onlay bone grafts, membrane techniques, bone distraction, and ridge splitting [3].

Dr. Hilt Tatum 1970s introduced a method of ridge splitting or bone spreading using specific instruments like D-shaped graduated osteotomes/wedges and tapered channel formers [4]. Later, Summers [5], Scipioni et al. [6] in 1994, and Sethi and Kaus [7] in 2000 revived and published articles on edentulous ridge expansion with 97–98.8% implant survival rate for over 5 years. With the emergence of implant dentistry and introduction of micro saws, piezo saws, and specific ridge split osteotomy, this technique has become an integral part of implant dentistry, wherein primarily bone expansion techniques were indicated in regions of division bone volume and density of D3 or D4. Bone due to its dynamic viscoelastic nature, thinner ridges (<3.5 mm) can be expanded with better controlled instrumentation with less risk for fracture, trauma, and bone perforations. The softer the trabecular bone quality, the lower the elastic modulus and the greater the viscoelastic nature of the ridge. Therefore, the lower the density of the bone, the easier and more predictable the bone expansion [8].

Lateral ridge split technique is a way to solve the problem of the width in narrow ridges with adequate height. Simultaneous insertion of dental implants will considerably reduce the edentulism time. Dental implant placement in atrophic ridges with deficiency in the bone volume with onlay bone-grafting techniques (autografts/allografts) needs some time between bone grafting and dental implant insertion (3–6 months) and there is always the possibility of bone graft failure. Crest split augmentation technique with simultaneous implant insertion will reduce the time of edentulism treatment. Bone compression and increase in trabecular density are other advantages of this technique [9]. For creating split between the cortical plates, different osseous surgical tools such as hand instruments (chisels), rotary instruments (surgical burs, saws), and piezosurgery instruments have been used successfully [10].

The piezosurgery device produces specific ultrasound frequency modulation (22 000–35 000 Hz). The unit provides extreme precision and safety as well as micrometric cutting. Moreover, the device causes less bleeding during and after the operation and the healing process is shorter [11].

Thermodynamic effects in bone produced by bur were widely described in the literature [12–14]. But modern medical technology is still developing and in the last two decades the following gained more and more popularity: erbium-chromium: yttrium-scandium-gallium-garnet (Er,Cr:YSGG) and erbium: yttrium-aluminum-garnet (Er:YAG) lasers. These lasers operate in the infrared spectrum at a wavelength of 2.78 (Er,Cr:YSGG) and 2.94 (Er:YAG) *μ*m and show good absorption in water; hence, these lasers afford good results in bone surgery [15, 16].

Extremely important during bone surgery is temperature rise, which is key factor for osseointegration process. When preparing and placing implants into a bone tissue, a nontraumatic surgical technique is critical. The heat generated during the preparation of the implant site is a major factor influencing surgery failure [17].

Eriksson and Albrektsson [18, 19] showed that increasing the temperature of the bone tissue by 10°C for 60 seconds induces permanent changes in the bone structure; therefore, the tissue temperature gradient (ΔTa) below 10°C should be considered optimal and safe.

*Objective*. To the authors best knowledge, thermodynamics effects during alveolar ridge splitting were not described in the literature. The aim of the study was to evaluate temperature gradient on pig model during ridge splitting by means of Er:YAG laser, piezosurgery unit, and surgical saw. Additionally, time of ridge splitting procedure and carbonization occurrence were assessed.

## 2. Materials and Methods

### 2.1. Samples Preparation

The research included 60 mandibles (*n* = 60) of recently slaughtered pigs (breed: Złotnicka Biała) intended for consumption and which had been obtained from a butcher. The skin of each mandible in the area between incisor (I1) and first molar (M1) tooth was cut off. The specimens were randomly divided in 4 groups (*n* = 15) according to the ridge splitting method and then were washed under the tap water and left for 4 hours before the research was commenced. In every specimen, preparation of the soft tissues in region of canine (C) and first molar (P1) tooth gave access to the buccal and lingual part of the mandibular alveolar ridge. The specimens after preparation were placed motionless in a clamp. The ethical approval was not required for this animal ex vivo study.

### 2.2. Surgical Procedure

**Figure 1:**
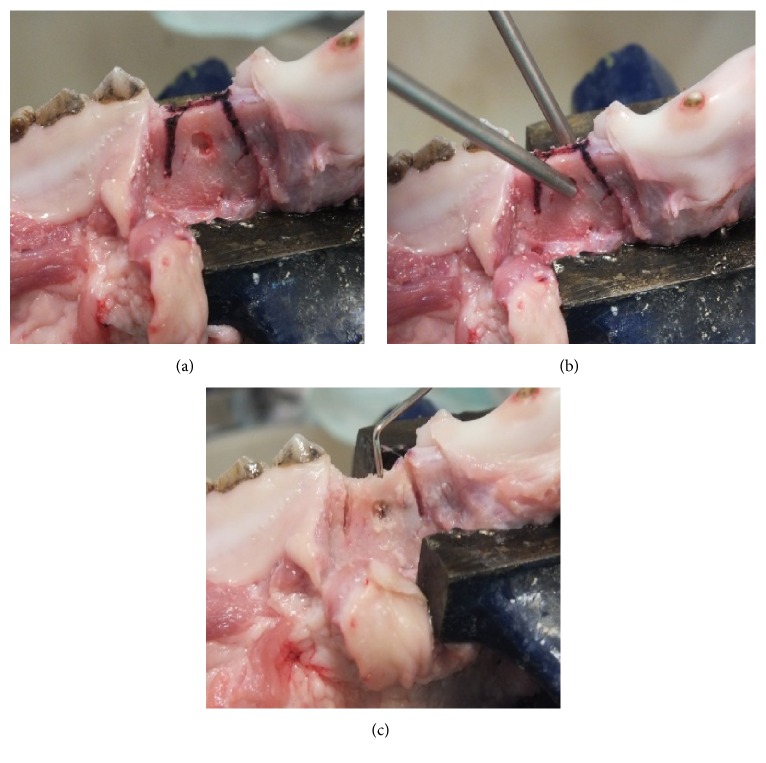
Monitoring and measurement of reaction to changes in temperature of the bone. (a) An alveolar ridge with cutting marks. (b) The thermocouples attached to the bone. (c) The control of osteotomies depth with a periodontal probe.

In the study area of the mandible a ridge splitting procedure has been done by two vertical cuts, 1 cm in length on the buccal side and 1 horizontal cut on the alveolar ridge 1 cm in length and 1 cm in depth by means of Er:YAG laser (LiteTouch®, Syneron Dental, Yokneam, Israel), piezosurgery unit (Piezotome Solo, Acteon, New Jersey, USA), and a saw disc (Hager & Meisinger GmbH, Hansemannstr., Germany) for a high-speed contra-angle hand piece (Intra C09-C3 27:1 Kavo, Biberach, Germany). In the buccal and lingual area of mandible 2.5 × 2.5 mm holes were made in the bone with a ball-shaped diamond bur for a high-speed contra-angle hand piece (Intra C09-C3 27:1 Kavo, Biberach, Germany) operated with a physiodispenser (Intrasurg300®, Kavo, Biberach, Germany) for temperature measure (Figure 1).

### 2.3. Measurement Procedure

The specimens were placed in a container with water at a temperature of 22°C for 20 minutes; the temperature was monitored with a Medicare Clinical Products (MCP) Gold mercury thermometer (Medicare Products Inc., New Delhi, India). The temperature of the bone was measured by means of a calibrated digital Thermocouple Meter, TM-902C thermometer (Zhangzhou Weihua Electronic Co., Fujian, China) with the temperature probe of the K, Thermocouple Probe, TP-02 type (Zhangzhou Weihua Electronic Co., Fujian, China). The measurement error was 0.75%. The temperature was measured in a continuous manner by means of probes attached in the central point of the prepared bone holes on the buccal and lingual side of the mandible. The highest difference of the bone temperature was recorded. The time of the bone preparation was measured with a sports stopwatch SP17 XL-009A (Fuzhou Swell Electronic Co., Ltd, Fujian, China).

### 2.4. Study Groups

The study specimens (*n* = 60) were divided into 4 groups: G1 (*n* = 15), G2 (*n* = 15), G3 (*n* = 15), and G4 (*n* = 15).

G1 group: Er:YAG laser (LiteTouch, Syneron Dental, Yokneam, Israel), operation mode for hard tissues (HT), was used, power: 200 mJ, frequency: 30 Hz, energy density per pulse: 15.07 J/cm^2^, water spray cooling (100%): 14 mL/min., tip angle set at 70°, size of the tip: 1.3 × 6 mm, and distance: 10 mm.
G2 group: Er:YAG laser (LiteTouch, Syneron Dental, Yokneam, Israel), operation mode for hard tissues (HT), was used, power: 400 mJ, frequency: 19 Hz, energy density per pulse: 30.14 J/cm^2^, water spray cooling (100%): 14 mL/min., tip angle set at 70°, size of the tip: 1.3 × 6 mm, and distance: 10 mm.
G3 group: piezosurgery unit (Piezotome Solo, Acteon, New Jersey, USA) was used: the parameters of the piezosurgery: tip: BSlS (cortical bone), CS1 (cutting depth), power: D1, and water spray cooling: 20 mL/min.
G4 group (control): tip: saw disc (Hager & Meisinger GmbH, Hansemannstr, Germany), saw diameter: 10 mm, speed: 1000 rpm, and water spray cooling: 20 mL/min.

#### 2.4.1. Statistical Analysis

The statistical analysis was performed by means of ANOVA variance analysis and *t*-test with the use of the programme Statistica 12 (StatSoft, Krakow, Poland) with free 30-day trial license. Values below *P* = 0.05 were considered to be statistically significant.

## 3. Results

An analysis of temperature gradient on bone surfaces revealed significant higher rise for piezosurgery (G3) on both lingual and buccal sides of an alveolar ridge as compared to Er:YAG laser (200 mJ, 400 mJ) and a saw (G4) (Table 1). The mean bone temperature increases during osteotomies using surgical saw were lower than in cases when the Er:YAG laser and piezosurgery were used. Furthermore, the temperature gradient measured in the lingual region of the mandible was significant lower as compared to the buccal part for each group. The bone cutting by means of piezosurgery caused much more temperature increases in the lingual region of a mandible even when comparing with an Er:YAG laser with energy set of 200 mJ and a saw on the buccal side.

**Figure 2:**
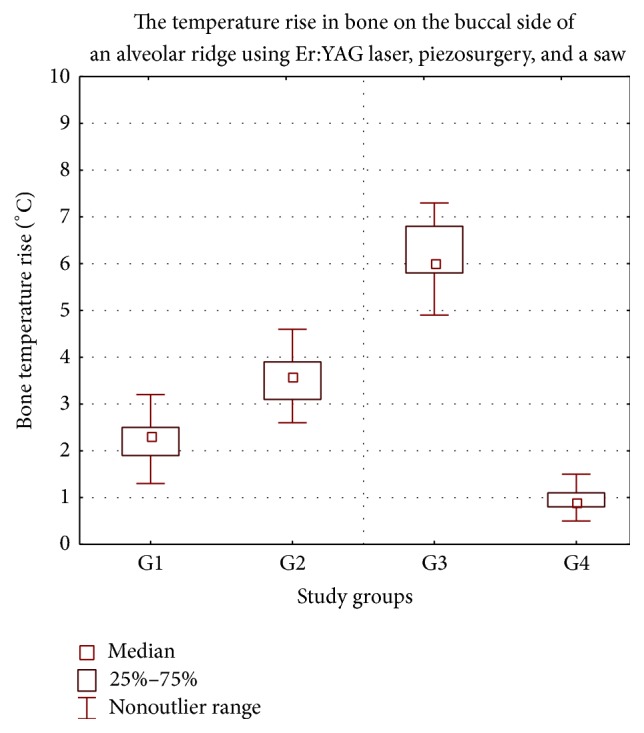
Increase in the temperature of the bone prepared with laser and saw in the buccal side of an alveolar ridge of a mandible. G1 (Er:YAG 200 mJ), G2 (Er:YAG 400 mJ), G3 (piezosurgery), and G4 (saw). °C: Celsius grade.

A significant bone temperature increase was observed following Er:YAG laser irradiation and a piezosurgery operation as compared to the saw in the buccal area of the mandible (Figure 2). Depending on the cutting device used, significant differences in bone temperature rise on the lingual side of an alveolar ridge between each group were also observed. Our findings show that following bone cutting with the Er:YAG laser, piezosurgery, and saw, the bone temperature on buccal side increased much more rapidly than it measured on the lingual side.

**Figure 3:**
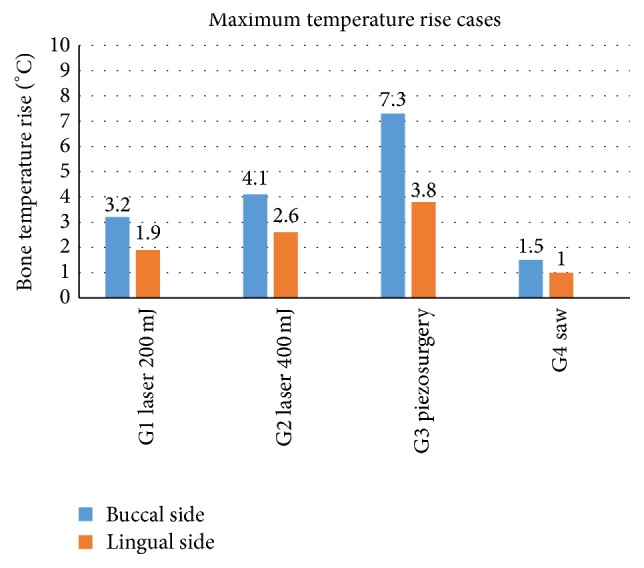
The highest results in temperature increase measured on the buccal and lingual side of an alveolar ridge of a mandible. °C: Celsius grade.

The maximum bone temperature of 7.3°C was noted for a specimen prepared using piezosurgery device (Figure 3). Additionally, the maximum temperature of none of the mandible rose by more than 10°C when applying different devices used in this study. Furthermore, the bone temperature after irradiation with an Er:YAG laser for energy of 400 mJ raised more quickly in comparison with the cases of energy equal 200 mJ.

The analysis of the ridge splitting time revealed significant differences in time needed for the bone osteotomies using Er:YAG laser and a piezosurgery as compared to the saw. Furthermore, significant differences in ridge splitting time depending on the cutting device used were also observed (Table 2). The time needed to perform a ridge splitting following an Er:YAG laser with power of 200 mJ and 400 mJ was 3- and 2-times longer as compared to the saw, respectively.

We also observed no signs of carbonization occurrence during bone cutting by means of Er:YAG laser, piezosurgery, and surgical saw.

## 4. Discussion

To the best of our knowledge, the comparison of Er:YAG laser, piezosurgery device, and saw on contra-angle for ridge bone splitting has not been discussed in the literature.

Heat is defined as a process in which energy flows from hot to cold objects. Despite the simple definition, heat transfer is an extremely complex physical phenomenon to analyze. A great deal of research has been expended to measure heat production during bone cutting with different techniques. Several important issues arise when dealing with temperature recording in bone tissue concerning the measuring device, the distance of the thermometer probe from the heat source, the cooling system, and the thermal properties of bone (e.g., type and shape of bone samples, thermal conductivity, and heat capacity). To overcome the limits related to the large number of factors at stake, a proper methodological approach and dedicated technical environment are essential [20].

In 2011, Rashad et al. [21] and Esteves et al. [22] prepared implant bed using two different ultrasonic devices (Piezosurgery, Mectron Medical Technology and VarioSurg, NSK) and one conventional drill. Result of their research showed that the heat production and time required for implant site preparation using both ultrasonic devices were significantly higher than those for conventional drilling (*P* < 0.01). Our study showed similar results for piezosurgery compared to the surgery saw during bone osteotomies.

Moreover, Agrawal et al. [23] suggested piezoelectric devices advantage over traditional methods of alveolar bone splitting due to the factors such as micrometric bone cut, clear surgical field, and selective cut. The authors emphasized that the piezosurgery device which operates with modulated ultrasound micro movements with oscillating frequency from 29 to 32 kHz, making it specifically suitable for osteotomies but not for a soft tissue cutting. According to researchers maximum surgical visibility is allowed during osteotomy, thanks to cavitation effect of the sterile saline. Stübinger et al. [24] also underlined advantage of piezoelectric unit over conventional rotary instruments in ridge splitting osteotomy. The authors drew attention to the biological aspects associated with the use of this type of devices. In their opinion one of the main benefits of using piezosurgery is reduced blood loss which improves healing conditions. Furthermore the constant irrigation helps to reduce thermal damage and thus reduces the risk of bone necrosis.

However, histopathological examination carried out by the Esteves et al. [22] on the rat bone exposed to create defects of 2 mm in diameter by using piezosurgery (piezo group) and conventional drilling (drill group) revealed that bone healing was similar in both groups with the exception of a slightly higher amount of newly formed bone observed at 30 days after surgery (*P* < 0.05). Ma et al. [25] in their studies reached similar conclusions. The purpose of their study was to compare bone healing of experimental osteotomies applying either piezosurgery or two different oscillating saw blades in a rabbit model. Authors claimed that all three osteotomy techniques revealed an advanced gap healing starting after one week. The most pronounced new bone formation took place between two and three weeks, whereby piezoelectric surgery revealed a tendency to faster bone formation and remodeling.

Our study which has taken into account an increase in temperature of the bone also has demonstrated the advantage of using piezosurgery as compared to conventional methods based on the rotary instruments in bone surgery.

In 2015 Rashad et al. [26] showed different result in comparison with temperature rise among sonic, ultrasonic, and conventional drills. Results of their newer findings were associated with lower heat generation compared to the conventional saw osteotomy. Copious irrigation seems to play a critical role in preventing heat generation in the osteotomy site. Lamazza et al. [27] described temperature gradient rise during piezoelectric implant bed preparation. Their study showed temperature gradient increase lower by 10°C after one minute of piezosurgery working. Our findings showed similar results.

Pandurić et al. [28] compared an Er:YAG laser (pulse energy, 1,000 mJ; pulse duration, 300 *μ*s; frequency, 20 Hz) and surgical drill for osteotomy in oral surgery. The temperature rise and time were assessed in their study. The Er:YAG laser removed significantly more bone tissue than the drill (*P* < 0.01) in a significantly shorter time (*P* < 0.01). Also the temperature was statistically lower during the laser preparation. Results are different than our findings. In another in vitro study on mandibular bones irradiated by an Er,Cr:YSGG laser Kimura et al. [29] stated that a temperature rise over 10°C (ΔTa) could be recorded 30 s after laser application. In our study a temperature rise over 10°C has not been recorded.

Romeo et al. [30] compared the peripheral bone damage induced by different cutting systems. The Er:YAG laser, piezosurgery, and high-speed and low speed drill have been utilized in their research. Four different parameters were analyzed: cut precision, depth of incision, peripheral carbonization, and presence of bone fragments. All sections obtained with the Er:YAG laser showed poor peripheral carbonization. The sections obtained by traditional drilling also showed poor peripheral carbonization. Piezosurgery incisions showed superficial incisions without thermal signs but with irregular margins. The level of carboxylation was evaluated with an optical microscope. The results of our study were similar. Making the optical evaluation by sight, we have not found carbonization in tested samples.

Results of our findings are also in coinciding with studies conducted by Lewandrowski et al. [31]. They compared the interaction of Er:YAG laser and traditional saw on the bone tissue. Based on the assessment of collected histological samples, they concluded that extent of thermal damage at the osteotomy sites was comparable for laser and mechanically saws.

Yoshino et al. [32] also in their study showed no severe thermal damage for Er:YAG laser compared to electrosurgery. Er:YAG laser irradiation without water coolant easily ablated bone tissue, and thermal alteration in the treated surface was minimal. In our studies for all specimens the thermal damage and carbonization of the bone have been not reported. Also results of lack of or minimal thermal damage in their research were similar to those of Martins et al. [33], Papadaki et al. [34], and Li et al. [35].

Stübinger [36], exchanging basic clinical benefits of using Er:YAG laser, stressed that in contrast to conventional osteotomy an Er:YAG laser enables noncontact interventions, no mechanical vibration, free and elaborate cut geometries, and aseptic effects. Passi et al. [37] also appreciated clinical benefits of Er:YAG laser in bone surgery compared to traditional drill method. Their study comprised 40 subjects requiring removal of impacted mandibular third molar, randomly categorized into two equal groups of 20 each, who had their impacted third molar removed either using Er:YAG laser or surgical bur as per their group, using standard methodology of extraction of impacted teeth. In next step they evaluated clinical parameters such as bleeding, pain, time taken for bone cutting, trismus, postoperative swelling, wound healing, and complications. Their study found that clinical parameters like bleeding, pain, and swelling were significantly lower in laser group than in the bur group. Wound healing and complications were assessed clinically and there was no significant difference in both the groups. Additionally, the laser group required almost double the time taken for bone cutting than bur. Stübinger et al. [38] demonstrated similar mind about the runtime of bone surgery using the Er:YAG laser. They emphasized that laser osteotomy was time-intensive and offered no depth control, and therefore it demonstrated only slight advantages for intraoral bone-grafting technics.

In our study, the time needed to carry out the alveolar ridge splitting on an animal model was also the highest for the laser group.

Many surgical techniques were adopted for a bone extension procedure. The bone ridge splitting is a surgical technique included an implant placement and guided bone regeneration in 1 stage. The ridge expansion technique in 1 stage was suggested as an alternative to horizontal and vertical augmentation techniques. The ridge splitting technique and osteodistraction are considered efficient to increase bone width with lower failure rate [39, 40]. The use of the osteotomes in a less dense bone (D3, D4) allows fracturing of bone trabeculae [41, 42]. However, this technique does not ameliorate peri-implant bone density. It was shown by Büchter et al. [43] that fractured trabeculae in a peri-implant bone, caused using the osteotome technique, induce a delayed secondary stability in comparison with conventional drilling protocol.

After alveolar ridge extension using saws and screw type osteotomes some proportion of bone undergoes necrosis due to interruption of the Havers' and Volkmann's canals. Vascular interruption, caused by drilling and cutting the bone, leads to necrosis of the osteocytes and thus to bone devitalization [44]. Also a temperature increase in bone over 10°C leads to death of osteocytes and to bone necrosis; thus, precise knowledge concerning the heat generation induced by laser, piezosurgery device, and a saw seems to be key factor of therapeutic success after ridge splitting procedure. All these facts together make variable ridge extension protocols using modern bone cutting devices necessary.

The recent study's showed superiority of Er:YAG during bone surgery, as compared to diode, Nd:YAG, KTP lasers. Fornaini et al. [45] pointed out the lower increase for Er:YAG and higher for diode laser. Their ex vivo study showed that laser utilization gives no risks of dangerous thermal elevation to the tissues. The key factors for preparing the bone and soft tissue by use of Er:YAG lasers are type of laser (Gaussian distribution of energy), short laser pulses duration, low power of laser beam, fluid pumping technology (fluid pressure), time of emission of a laser beam, and type of laser tip. Er:YAG laser without optic fiber and with rectangular energy distribution profile generate high uniform power with regard to the beam and with low energy losses during transport. In most of the presently used lasers, the energy beam is transported to the tip by means of an optic fiber, which distorts the energy distribution. In such lasers, the highest energy is located only in the middle of the beam and it is much lower at the edges. Concentration of the beam power in the very centre (older technology) with relatively low power and high frequency settings may cause thermal damage in the bone. A new laser technology results in reductions of Er:YAG laser defects, for example, overheating and carbonization [46, 47].

Furthermore in our study the Magnum tip 1.3 mm in diameter and 6 mm in length was used. This is the only one tip which allows transferring laser light without its defocusing. Therefore this tip does not change laser beam distribution of energy from flat-top to Gaussian profile as compared to glass optic fiber. Hence, higher energy density irradiated target tissue in shorter time causes less thermal damage. Thus, quantity of energy irradiated on the target area is the most productive and ablation of the tissue is fast and cuts are clean without carbonation effects. Further studies should be conducted to establish predictable and safe clinical protocol of different procedures in laser surgery. Additionally, an influence of tip size on temperature change in the bone should be evaluated.

## 5. Conclusions

The Er:YAG laser has great potential in advancing surgical techniques where precision in osseous preparation is required. Piezosurgery, Er:YAG laser, and surgical saw are useful and safe tools in ridge splitting surgery. For all devices the temperature rise was below 10°C, which confirmed safeness and predictability of these methods.

## Figures and Tables

**Table 1:** Mean temperature gradient and standard deviation data measured in buccal and lingual sides of an alveolar ridge.

Variable	ΔTa (°C) ± SD buccal	ΔTa (°C) ± SD lingual	*P* value (buccal versus lingual area)
Group 1 (*n* = 15)	2.23 ± 0.47	1.19 ± 0.49	0,0000021
Group 2 (*n* = 15)	3.49 ± 0.54	2.09 ± 0.27	0,0000927
Group 3 (*n* = 15)	6.19 ± 0.70	3.17 ± 0.35	0,0000775
Group 4 (*n* = 15)	0.93 ± 0.27	0.53 ± 0.21	0,0000966

**Table 2:** Mean time required to perform mandibular ridge splitting. The results showed significant differences in comparison with ridge splitting time in comparison between each group, respectively (*P* < 0.05).

Study groups	Time *t* (sec) ± SD
Group 1 (*n* = 15)	538.47 ± 70
Group 2 (*n* = 15)	360.73 ± 58.03
Group 3 (*n* = 15)	305.13 ± 54.84
Group 4 (*n* = 15)	172.07 ± 41.56
*P*	<0.05
